# Phenotypic Resistance to Antibiotics

**DOI:** 10.3390/antibiotics2020237

**Published:** 2013-04-18

**Authors:** Fernando Corona, Jose L. Martinez

**Affiliations:** Centro Nacional de Biotecnología, CSIC, Darwin 3, 28049-Madrid, Spain

**Keywords:** phenotypic resistance, biofilm, persistence, bacterial permeability, antibiotic resistance, resistome

## Abstract

The development of antibiotic resistance is usually associated with genetic changes, either to the acquisition of resistance genes, or to mutations in elements relevant for the activity of the antibiotic. However, in some situations resistance can be achieved without any genetic alteration; this is called phenotypic resistance. Non-inherited resistance is associated to specific processes such as growth in biofilms, a stationary growth phase or persistence. These situations might occur during infection but they are not usually considered in classical susceptibility tests at the clinical microbiology laboratories. Recent work has also shown that the susceptibility to antibiotics is highly dependent on the bacterial metabolism and that global metabolic regulators can modulate this phenotype. This modulation includes situations in which bacteria can be more resistant or more susceptible to antibiotics. Understanding these processes will thus help in establishing novel therapeutic approaches based on the actual susceptibility shown by bacteria during infection, which might differ from that determined in the laboratory. In this review, we discuss different examples of phenotypic resistance and the mechanisms that regulate the crosstalk between bacterial metabolism and the susceptibility to antibiotics. Finally, information on strategies currently under development for diminishing the phenotypic resistance to antibiotics of bacterial pathogens is presented.

## 1. Introduction

Most studies on the development of antibiotic resistance deal with inheritable resistance. Under this view, the acquisition of a resistance phenotype requires a genetic change; either mutations (including point mutations, deletions and insertions), or the acquisition by horizontal gene transfer of antibiotic resistance genes [1,2,3,4,5]. However, other situations in which bacteria become transiently resistant to antibiotics, in the absence of a genetic change, have been also described [6]. Among them, the most studied are drug indifference, the growth in biofilms, and the phenomenon of persistence. However, there are other situations wherein bacteria present changes in their susceptibility to antibiotics depending on their metabolic state. Whilst this phenotype of resistance to antibiotics has been in occasions attributed to a situation of growth arrest that precludes the activity of some bactericidal antibiotics as beta-lactams, a growing number of evidences indicates that the link between the metabolic state of bacterial populations and their phenotype of susceptibility to antibiotics is far more complex. Although increased resistance can also be achieved because of the induction of specific resistance mechanisms (for instance induction of chromosomally-encoded β-lactamases by β-lactams [7]), this topic is not usually considered as phenotypic resistance and will not be discussed in the article.

Some recent works have analyzed the determinants that contribute to the intrinsic resistance (intrinsic resistome) of bacterial pathogens [8,9,10,11,12,13,14,15,16]. Notably, in all cases in which a comprehensive study has been performed the number of genes involved in the phenotype of resistance is larger than could be predicted if they had evolved as specific elements for counteracting the action of the drugs. Furthermore, several of such genes encode key elements of the bacterial metabolism. Altogether, these results indicate that the specific phenotype of susceptibility to antibiotics is under metabolic control and hence that changes in the bacterial metabolism can consequently alter the susceptibility to antibiotics [17,18]. 

The fact that the susceptibility to antibiotics can change depending on the bacterial metabolic state is a two-way road. Bacteria can be transiently more resistant to antibiotics, a situation that may compromise therapy. Conversely, there can be specific metabolic conditions that increase the susceptibility to antibiotics. This situation has been recently exploited for developing nutritionally-based strategies for fighting persistent cells [19]. Similarly, the comparison of the metabolism of *Mycobacterium tuberculosisin vitro* and *in vivo* has served to understand the reasons why some lead compounds with very good activity *in vitro* and good pharmacological properties were not useful in a tuberculosis model of infection. The understanding of the causes of this phenotypic resistance, which is due to differences in the carbon metabolism of *M. tuberculosisin vivo* and *in vitro*, is important for the development of novel anti-tuberculosis antibiotics [20].

Several articles have reviewed the acquisition of antibiotic resistance determinants by human pathogens. However, information on phenotypic resistance is lower in comparison. In the current article, some specific examples of situations that produce a transient, non-inherited, state of antibiotic resistance are reviewed. 

## 2. Drug Indifference

**Figure 1:**
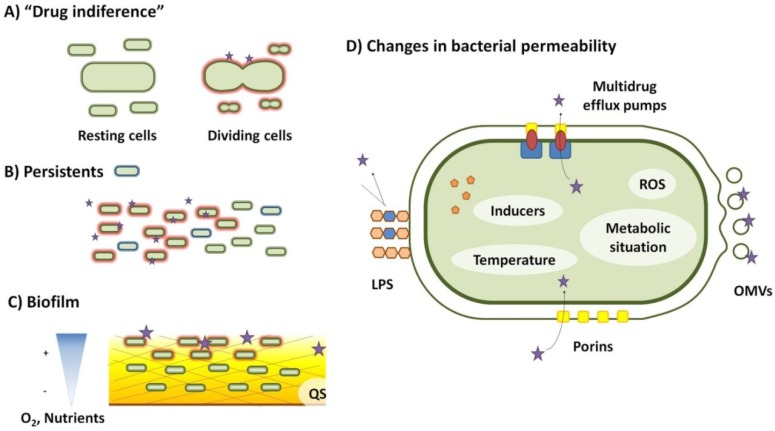
Some situations of transient changes of bacterial susceptibility to antibiotics are described. (**A**) Drug indifference occurs when the antibiotic is effective only in a specific bacterial physiological condition. This situation was first described for β-lactam antibiotics that do not kill non-dividing growing cells; (**B**) In a bacterial population, there is a subpopulation of cells called persistents that are not killed by antibiotics in conditions that kill the bulk of the population. Once growth resumes, these persistent bacteria become susceptible to antibiotics, indicating that the phenotype of resistance is transient, not being the consequence of a genetic change; (**C**) Bacteria usually grow forming biofilms when attached to surfaces and under these conditions they are more resistant to antibiotics. Gradients of nutrients and of oxygen cause different metabolic states in the bacteria depending on their depth inside the biofilm, which can affect their susceptibility to antibiotics. Moreover, compounds of the matrix can impair the diffusion of the antibiotic and eventually bind the drug thus reducing its free concentration. Quorum sensing, which can be triggered at dense regions of the biofilm, may also alter bacterial susceptibility to antibiotics. (**D**) Bacteria change their permeability to antibiotics in response to several environmental and internal factors such as temperature, the presence of specific inducers, reactive oxygen species (ROS) or specific metabolic situations. Changes can affect antibiotic susceptibility at several levels. First, reducing the binding of the antibiotic by modification of the lipopolysaccharide (LPS), or generating outer membrane vesicles that give rise to more surface and minimizes the effective amount of the antibiotic per cell. Second, modifying the number or type of bacterial porins, the aqueous channels used by the antibiotic to penetrate inside bacteria, might help keeping the antibiotics out of the cell. Third, expressing multiple efflux pumps that extrude the antibiotic once it has reached the cytoplasm decreases the toxic activity of the antibiotic.

Soon after the introduction of antibiotics for treating infections, it was described that resting cells are less susceptible to penicillin [21], a situation (Figure 1) that was named ‘drug indifference’ [22]. Some recent works have shown that this effect is specific for the type of antibiotic considered. Non-dividing cells are fully resistant to ampicillin and tetracycline, whereas ciprofloxacin and streptomycin are active against stationary cells although their level of activity is lower than when cells are actively growing [6].

The reduced susceptibility to antimicrobials that depends on the growth rate can be relevant for bacteria growing at host locations where growth is restricted or in situations where the bacteria have made use of the nutritional resources provided by the host and hence growth rate is reduced. This might be particularly relevant for long-lasting infections. In fact, it has been described that the antibiotic concentrations required for curing an infection are directly related to the duration of the infection [23]. Further works using a model in which mice were infected with a mixture of susceptible and resistant bacteria showed that during infection bacteria become increasingly refractory to treatment [24]. This reduced susceptibility is not inheritable and is the consequence of changes in the bacterial physiology. More recently, it has been shown that eight hours after infection bacteria are in a resting situation [6], which can cause the observed drug indifference, at least in this experimental model of infection.

These finding are of relevance for the treatment of human infections. It has been suggested that the existence of drug indifferent, slow growing (or resting) cells can be the cause of relapse after antibiotic treatment that can be observed for some bacterial infections [25,26]. It has been stated as well that this low susceptibility is the cause for the need of using very long treatments for slow-growing bacteria as *Mycobacterium leprae* [27] or *Mycobacterium tuberculosis* [28]. Finding drugs that can act on these ‘drug refractory’ cells is thus of interest for the treatment of long lasting infections or infections due to slow-growing pathogens. An example of this view is the potential use of antibiotics that specifically kill anaerobes for the treatment of infections by *M. tuberculosis*, since it has been described that dormant, hypoxic *M. tuberculosis* cells are resistant to isoniazid, but susceptible to metronidazol [29,30,31]. 

## 3. Biofilms

Although most studies on bacterial susceptibility to antibiotics are performed using bacteria growing planktonically, microorganisms can grow forming biofilms when they are attached to a surface [32,33,34,35]. For human pathogens, this situation can be particularly relevant in the case of chronic infections of prosthetic devices. Different works have shown that bacteria forming biofilms are less susceptible to antibiotics than those growing planktonically [36,37,38,39,40,41,42,43]. Furthermore, it has been described that subinhibitory concentrations of antibiotics can trigger the formation of biofilms by bacterial populations [44,45], suggesting that antibiotics can induce transient, non-specific resistance to themselves. There are several potential reasons why biofilms are more resistant to antibiotics (Figure 1). The most obvious one is that biofilms are complex structures in which the free diffusion of compounds as antibiotics [46,47] is more difficult that in liquid cultures (planktonic cells). The capability to diffuse into the biofilm will differ depending on the structure of the antibiotic [48]. Increasing the diffusion of the antibiotics inside biofilms might thus help in enhancing their activity [49]. However, this would be just a part of the problem [50]; in several occasions the speed in the penetration of the antibiotics into the biofilm does not seem to be the key element for explaining the phenotype of resistance [51]. The biofilm structure itself may contain elements that bind antibiotics, sequestering them and hence reducing the freely available concentration of these compounds within the biofilm structure. 

More recent articles have focused on the metabolic state of bacteria when growing forming biofilms. It has been suggested that the biofilm-forming microorganisms can display different metabolic situations, including actively growing cells that should be killed by antibiotics and resting cells that would be transiently resistant to these compounds. These resting cells might be a subpopulation of persisters (see below) present in any biofilm [52]. The different metabolic situation of each subpopulation is highly dependent on the oxygen and nutrients availability, which is higher at the upper layers of the biofilm and lower at the deeper ones [53,54]. Surprisingly, the effect of these metabolic situations is different depending on the antibiotic family. It has been found that the aerobic regions of *Pseudomonas aeruginosa* biofilms are susceptible to quinolones and resistant to cationic peptides whereas the opposite occurs at the hypoxic regions [55]. One of the potential causes of the resistance to cationic peptides of biofilms relies on their structure itself. One of the compounds forming the biofilm extracellular matrix is DNA. This macromolecule chelates cations, and reduced concentrations of divalent cations trigger expression of the master sensor regulator of resistance to cationic peptides PhoP-PhoQ [56]. It has been then suggested that the use of DNase, which can break the biofilm matrix, may increase biofilm susceptibility to antibiotics [57]. In addition to the effect of metabolic changes on the susceptibility to antibiotics to biofilm-growing bacteria, classical resistance determinants may also be involved in the phenotype of resistance. For instance, the efflux pump MexAB-OprM contributes to the lack of susceptibility of *P. aeruginosa* biofilms [58], whilst it is not relevant in the case of planktonic cells. 

Altogether, these results indicate that there are several, simultaneous mechanisms that alter the susceptibility to antibiotics of bacteria growing in biofilms. Understanding the signals and mechanisms involved in the formation of biofilms can thus help in eliminating them. In the case of *P. aeruginosa*, the quorum sensing (QS) response triggers biofilm formation. Inhibition of QS thus reduces the formation of biofilm and consequently increases susceptibility to antibiotics. One of the compounds that inhibits biofilm formation is the macrolide azithromycin [59]. Although Gram-negative bacteria as *P. aeruginosa* are intrinsically resistant to macrolides, azithromycin has been proposed as a drug of choice for the treatment of chronic *P. aeruginosa* infections given its strong anti-QS and anti-biofilm activity [60,61].

## 4. Transient Changes in Bacterial Permeability to Antibiotics

One of the main factors in the effectiveness of the antibiotics is their penetration inside the bacterial cell. This means that the first barrier against antibiotics consists on the bacterial envelopes. Consequently, changes in bacterial permeability may affect the susceptibility to antibiotics [62,63]. The permeability to antibiotics of microorganisms can be modulated by means of three different mechanisms (Figure 1). First, bacteria may alter their surface by modifying the lipopolysaccharide, reducing the molecular interactions with the antibiotic, and consequently, its penetration. Production of membrane vesicles increases the available membrane surface, which can also reduce the effective concentration of antibiotic available to enter the cell. Second, alterations in the number or type of porins or antibiotic transporters may change the susceptibility to antibiotics. Third, once the antibiotic reaches the cytosol, it can be pumped actively outside the cell through efflux pumps. 

In Gram-negative bacteria, the first barrier to antibiotic penetration is the outer membrane, mainly composed in its outer face by the lipopolysaccharide (LPS). The LPS presents anionic groups where the cationic antibiotics bind, representing the first step of antibiotic uptake [64]. 

In *P. aeruginosa*, it has been described that the alteration of the negative charge of the lipid A of the LPS by adding 4-aminoarabinose, increases the resistance to polymyxin, aminoglycosides and cationic antimicrobial peptides. This response can be induced by limiting concentrations of the divalent cations Mg^2+^ and Ca^2+^, or of polymyxin and cationic antimicrobial peptides. In *P. aeruginosa*, there are at least three two-component systems (PhoP-PhoQ [65,66], PmrA-PmrB [67], and ParS-ParR [68]) that upregulate the arnBCADTEF operon, whose products are responsible for the changes in the LPS. The induction of resistance to cationic antibiotics by environments with low amounts of Mg^2+^ has been also described in *Salmonella typhimurium* [69]. Especially relevant in the clinic is the induction of antibiotic resistance by host-defense peptides belonging to innate human defense [70], and for the new peptides that are being developed to be used in the frame of novel anti-infective therapeutic strategies [71].

A similar phenomenon of LPS modification leading to transient resistance to aminoglycosides has been described in *Stenotrophomonas maltophilia* [72]. The authors found a correlation between the LPS pattern and an increase in the resistance to aminoglycosides when the bacteria were grown at 30 °C. Although the molecular basis of transient induction of resistance in such conditions has not been clarified, these results strongly suggest that the growth temperature affects the LPS composition challenging the binding or the uptake of aminoglycosides.

Another mechanism that might transiently induce resistance in Gram-negative microorganisms is the formation of Outer Membrane Vesicles (OMVs). It has been proposed that OMVs can trigger an initial defense mechanism against different environmental stresses including antibiotics [73]. OMVs are made with fragments of the bacterial outer membrane enclosing periplasm and active proteins. Among the roles that OMVs might play, it has been suggested that they are involved in secretion, intercellular signal trafficking and in bacterial survival-related functions [74]. Like LPS, OMVs can bind cationic peptides and antibiotics, which suggests that they can provide transient resistance to these compounds just by a trapping mechanism that reduces the free concentration of the drug [73]. It is known that some factors like temperature and nutrient limitation can increase the production of OMVs. Furthermore, the composition and number of OMVs are different in biofilms and in planktonic cultures [73]. Whether or not induction of OMVs formation under such environmental conditions can induce transient antibiotic resistance is still an open question that merits to be explored.

To enter into bacterial cells, antibiotics can make use of regular transporters (porins and inner membrane transporters) used for the microorganisms to allow the entrance of different substrates, including nutrients [63,75]. *Escherichia coli* harbors two main porins, OmpC and OmpF. These two porins are involved in the transport of some antibiotics such as quinolones, tetracycline, chloramphenicol or β-lactams. Low-level expression or inactivation of porins impairs the intracellular accumulation of the antibiotic and consequently renders resistance [75]. This implies that changes in the expression of porins due to environmental signals may alter bacterial susceptibility to antibiotics [62]. It has been described that OmpC and OmpF are inversely regulated. OmpF is highly produced in environments with low osmolarity and temperature, whereas OmpC is the prevailing porin when bacteria are growing under conditions of high osmolarity and high temperature, like for example when they are colonizing a nutrient-rich animal gut [76]. Since each porin has different substrates profile, the consequences of this regulation for antibiotic resistance will have some degree of specificity [75]. The expression of porins is finely tuned in response to changes in the environment; because of this, they are under control of complex regulatory networks. The phosphorelay system EnvZ-OmpR senses osmolarity and controls at the transcriptional level the OmpF/OmpC ratio [77,78]. On top of this regulation, there are two small antisense RNAs (*micF* [79] and *micC* [80]) which participate in the post-transcriptional regulation of OmpF and OmpC, respectively. *micF* is known to respond to changes in osmolarity [81], temperature [82] and nutrient availability [83], and is the link to other regulatory networks such as the Mar-Sox-Rob regulon, which responds to salicylate [84]. In this way, fast changes in the environment can be rapidly faced up by the microorganisms and this has consequences in bacterial susceptibility to antibiotics.

As above stated, multidrug efflux pumps can extrude a broad range of substrates chemically different, including antibiotics [85,86,87,88,89,90]. Genomic studies have shown that multidrug efflux pumps can account for 10% of all transporters in some bacterial species [91,92,93]. Together with their high conservation, this suggests that efflux pumps have important roles for the bacterial physiology in addition to being antibiotic resistance determinants [89,90]. Expression of efflux pumps is usually tightly down-regulated, which means that transient expression is achieved just in the presence of the right effectors [94,95]. Among those effectors that trigger expression of MDR efflux pumps, some might be relevant during infections. These include biocides such as triclosan, which is found in several products from soaps to toothpastes, and which has been shown to induce transiently the expression of the SmeDEF efflux pump from *S. maltophilia* [96]. More relevant from the therapeutic point of view is the induction of efflux pumps (and consequently resistance) in the presence of bile, cationic peptides or fatty acids [97,98,99,100,101], because bacteria can encounter these compounds and therefore might display a phenotype of transient resistance in the course of an infection. 

In addition to their induction by specific effectors, expression of efflux pumps can be altered by more general changes to their habitat. For example, it has been described that oxidative and nitrosidative stress [102] might alter the expression of multidrug efflux pumps. In *E. coli*, the exposure to oxidative agents like paraquat, which produces anion superoxide O^2−^, induces the SoxRS operon. As a consequence, the expression of the AcrAB-TolC multidrug efflux system is induced [103] and bacteria become transiently antibiotic resistant. In *P. aeruginosa*, another regulator, MexR, which is the repressor of *mexAB-oprM* MDR efflux pump, is directly oxidized by oxidative agents. As a consequence, MexAB-OprM is overexpressed leading to transient antibiotic resistance [104]. A similar response to oxidative and nitrosidative stress has been described for other *P. aeruginosa* efflux pumps, like MexXY-OprM [105], and MexEF-OprN [106]. Since bacteria can face oxidative and nitrosative stress during infection, it is likely that these efflux pumps are overexpressed and bacteria present a phenotype of reduced susceptibility to antibiotics in this situation. However, this topic has not yet been addressed in detail.

We have presented examples in which changes in membrane permeability trigger transient antibiotic resistance. However, the opposite might also happen. In some cases, *in vivo* growing conditions can lead to an increase in antibiotic susceptibility allowing a better response to anti-infective therapy. This is the case of susceptibility to fosfomycin of *Listeria monocytogenes* [107]. *Listeria* is an intracellular pathogen. *In vitro*, *Listeria* is resistant to fosfomycin. However, it has been shown that when growing inside the host cells *L. monocytogenes* becomes susceptible to this antibiotic. Fosfomycin enters in bacteria using Hpt, the same transporter as hexoses-phosphate and glycerol-phosphate [108]. The Hpt transporter is controlled by the global regulator of virulence, PfrA, which is highly expressed during macrophage infection [107,109]. One of the nutrients that *L. monocytogenes* uses at the intracellular milieu is glucose-phosphate. For an efficient uptake of this nutrient, the expression of its transporter Hpt is induced in intracellular bacteria, hence causing the observed increased susceptibility to fosfomycin. In this way, an antibiotic whose use has been discarded based on classic susceptibility tests, would have a potential use in clinics, to treat *Listeria* infections.

From these results, it is clear that the metabolic status of the microorganisms is of relevance for their susceptibility to antibiotics [17]. It is thus possible that global metabolic regulators may also modulate the antibiotic resistance. This is the case of Crc, a global post-transcriptional regulator of carbon metabolism in *P. aeruginosa* [110,111,112,113]. It has been described that in addition to regulating the utilization of alternative carbon sources in nutrient-rich environments, Crc modulates the virulence and antibiotic susceptibility of *P. aeruginosa* [18]. Bacterial transporters are targets for Crc. For example, the expression of the OprD2 protein, involved in the uptake of basic amino acids and of the antibiotic imipenem, and of GlpT, involved in the uptake of glycerol phosphate and of the antibiotic fosfomycin, are downregulated by Crc. As a consequence, a Crc defective *P. aeruginosa* mutant is more susceptible to these antibiotics. This makes Crc a good target in the search of drugs, to be used in combination, for enhancing the activity of antibiotics currently in use. 

## 5. Inoculum Size and Phenotypic Resistance

Several reports have shown that the size of the bacterial inoculum is important for the activity of antibiotics [114,115,116,117]. It has been suggested that this effect can be the cause of the failure for treating infections with a high bacterial load, because the actual minimal inhibitory concentrations of such bacterial populations are higher than those determined using classical laboratory tests [115,118]. In the case of bacteria producing antibiotic-inactivating enzymes, this effect is due to the activity of the enzyme that degrades the antibiotic at higher rates when more cells are present [116,117,119]. However, the situation is less clear in the case of bacteria that do not harbor a mechanism of resistance that involves the degradation of the antimicrobial. For these microorganisms and antibiotics, two situations can be envisaged. One is a reduction in the antibiotic concentration at high cell densities due to its binding to cell envelopes and debris of alive and killed cells. Another is the existence of less molecules of antibiotic per cell at high cellular density. Using experimental approaches and mathematical modeling, it has been shown that most likely both situations are relevant for inoculum-dependent antibiotic resistance [120], and that other processes can be important for this phenotype. Taken into consideration that high cell densities trigger the QS response and the fact that QS might affect the susceptibility to antibiotics it is worth considering that the QS response might have a role in the inoculum-dependent antibiotics susceptibility of bacterial populations. Nevertheless, to the best of our knowledge, this possibility has not yet been explored.

## 6. Persistence

Persistence to antibiotic treatment has been described for several different bacterial species, and consists (Figure 1) on a situation in which a subpopulation of the bulk of bacteria under treatment presents a refractory state to the action of antibiotics [121,122,123]. The relative fraction of cells in this situation can range from 10^−6^ for exponentially growing bacteria to 10^−2^ for bacteria in the stationary growth phase. Resistance is transient; persistent cells resume growth once the antibiotic is removed, but are killed at a similar rate as the original susceptible population if the antibiotic is added again [124]. For most infections, phenotypic resistance to antibiotics of a very small number of cells is not problematic, since these cells are removed by the action of the immune system. However, there are situations in which persistence can be a relevant problem for the success of the treatment of the infection.

One possible explanation for persistence is that, in any bacterial population, a fraction of the cells is in a non-dividing (dormant) situation that makes them refractory to the action of the antibiotics. However, the situation is more complex [125], with a number of global regulators and metabolic enzymes involved in the process [126,127]. Whilst bacterial cultures can contain a subpopulation of pre-existing dormant cells (Type I persisters), there is another subpopulation (Type II persisters) that emerges as the consequence of the inherent bistability of growing cells, from normal to persisters [121]. The main factor triggering Type I persistence is starvation. Nevertheless, the phenomenon is not a direct response to a non-growing situation of bacterial cells. On the contrary, the fact that Type I persisters are fully susceptible to antibiotics in a short time window after the beginning of growth of the whole population indicates that persistence consists on a metabolic shift that occurs after the stationary phase is over [128]. One of the key elements for the establishment of Type I persistence is the stringent response as shown by the relevant role that SpoT and RelA have for persistence in *E. coli* [129]. However, some different works have shown that there are different mechanisms triggering persistence, among them the inherent bimodal distribution of toxin/antitoxin proteins that can render non-uniform bacterial populations in which those cells containing high levels of toxin arrest their growth for long periods of time [39,130,131]. It is important to notice that infection-linked situations can trigger persistence. For instance, quinolones can induce persistence in *E. coli* through the SOS-mediated induction of the toxin TisB [132] and intracellular pathogens can present non-dividing, persistent, subpopulations when growing inside their host cells [133].

The increased knowledge on the mechanisms of persistence has led to propose different approaches for eradicating these bacterial subpopulations that are refractory to antibiotic treatment. In the case of *P. aeruginosa*, it has been described that the QS response is relevant for persistence [134].

Inhibition of QS has been suggested as an anti-virulence approach for fighting *P. aeruginosa* infections [135,136]. Reduction of the fraction of persistent cells or enhancing their susceptibility to antibiotics by this kind of inhibitors will also be of benefit for the treatment of *P. aeruginosa* infections. Recent work has shown that the QS inhibitor (*Z*)-4-bromo-5-(bromomethylene)-3-methylfuran-2(5*H*)-one restores the antibiotic susceptibility of *P. aeruginosa* PAO1 persisters. However, the effect is not due to the inhibition of QS and the mechanisms for this activity remain to be fully determined [137].

Some works have shown that the knockout of genes coding for metabolic enzymes, such as *ygfA* that encodes an enzyme involved in folate biosynthesis or *yigB*, encoding a flavin mononucleotide phosphatase decreases persistence [126], whereas their overexpression, increases tolerance. Given that persistence can be under metabolic control, another approach for eliminating persisters is by shifting the bacterial metabolism. The feasibility of this approach has been demonstrated in a set of recently published articles. In one of them, the authors were able to kill persister cells with aminoglycosides just by adding specific metabolic substrates that allow the recovery of bacterial proton motive force without resuming growth [19]. Following the same approach, it has been found that the addition of 3-[4-(4-methoxyphenyl)piperazin-1-yl]piperidin-4-yl biphenyl-4-carboxylate to bacterial cultures causes the reversion of persisters to antibiotic-sensitive cells [138].

A recent article explored another possibility for eradicating persisters. It was observed that *M. tuberculosis* persisters require a small reduction in oxygen availability for their survival to antibiotics. At high concentrations of oxygen, persisters were killed at the same rate as normal cells. Since his situation could be a consequence of a reduced capability of persisters to generate reactive oxygen species (ROS) in the presence of bactericidal antibiotics, priming ROS generation will eliminate persisters. In line with this reasoning, the authors demonstrated that antibiotic clofazimine, which increases ROS, successfully eradicates the persister population [139].

## 7. Concluding Remarks

Most studies on the mechanisms of antibiotic resistance address the development of resistance as the consequence of a genetic, inheritable change, which can be a mutation or the acquisition of a resistance gene. These are off/on directional situations; bacteria are either susceptible or resistant and reversion from resistance to susceptibility is not a frequent event. Several works have shown that this is a part of the resistance landscape and that there are situations in which resistance is not driven by a genetic change. Transient, reversible resistance can be achieved by different mechanisms, which are linked to the physiological state of bacteria and to the inputs that microorganisms receive when confronted to different habitats and stressors. The study of the mechanisms of phenotypic resistance is of relevance to understand the reasons of therapeutic failures for bacteria that are classified as susceptible using classical testing methods, but also to increase the susceptibility to antibiotics of bacterial pathogens. 

Classical methods for improving the efficacy of antibiotics are mostly based on the development of permeabilizers and of inhibitors of mechanisms of resistance, mainly of antibiotic inactivating enzymes and of multidrug efflux pumps [9,140,141,142]. The study of the mechanisms leading to phenotypic resistance may also serve for this purpose. As stated in the review, this knowledge may serve, among other issues, to eliminate persister cells by priming their metabolism, to implement novel strategies for treating infections by intracellular pathogens, which are more susceptible to some specific antibiotics when growing inside the cell, to develop antimicrobials with anti-biofilm activity, or to define novel targets, which inactivation would increase the susceptibility to antibiotics.

## References

[1] Baquero F., Coque T.M. (2011). Multilevel population genetics in antibiotic resistance. FEMS Microbiol. Rev..

[2] Martinez J.L., Fajardo A., Garmendia L., Hernandez A., Linares J.F., Martinez-Solano L., Sanchez M.B. (2009). A global view of antibiotic resistance. FEMS Microbiol. Rev..

[3] Martinez J.L., Baquero F. (2000). Mutation frequencies and antibiotic resistance. Antimicrob. Agents Chemother..

[4] Boto L., Martinez J.L. (2011). Ecological and temporal constraints in the evolution of bacterial genomes. Genes.

[5] Baquero F., Alvarez-Ortega C., Martinez J.L. (2009). Ecology and evolution of antibiotic resistance. Environ. Microbiol. Reports.

[6] Levin B.R., Rozen D.E. (2006). Non-inherited antibiotic resistance. Nat. Rev. Microbiol..

[7] Wiedemann B., Pfeifle D., Wiegand I., Janas E. (1998). beta-Lactamase induction and cell wall recycling in gram-negative bacteria. Drug Resist. Updat..

[8] Fernandez L., Alvarez-Ortega C., Wiegand I., Olivares J., Kocincova D., Lam J.S., Martinez J.L., Hancock R.E. (2013). Characterization of the polymyxin B resistome of *Pseudomonas aeruginosa*. Antimicrob. Agents Chemother..

[9] Martinez J.L. (2012). The antibiotic resistome: Challenge and opportunity for therapeutic intervention. Future Med. Chem..

[10] Alvarez-Ortega C., Wiegand I., Olivares J., Hancock R.E., Martinez J.L. (2011). The intrinsic resistome of *Pseudomonas aeruginosa* to beta-lactams. Virulence.

[11] Fajardo A., Martinez-Martin N., Mercadillo M., Galan J.C., Ghysels B., Matthijs S., Cornelis P., Wiehlmann L., Tummler B., Baquero F. (2008). The neglected intrinsic resistome of bacterial pathogens. PLoS One.

[12] Schurek K.N., Marr A.K., Taylor P.K., Wiegand I., Semenec L., Khaira B.K., Hancock R.E. (2008). Novel genetic determinants of low-level aminoglycoside resistance in *Pseudomonas aeruginosa*. Antimicrob. Agents Chemother..

[13] Breidenstein E.B., Khaira B.K., Wiegand I., Overhage J., Hancock R.E. (2008). Complex ciprofloxacin resistome revealed by screening a *Pseudomonas aeruginosa* mutant library for altered susceptibility. Antimicrob. Agents Chemother..

[14] Liu A., Tran L., Becket E., Lee K., Chinn L., Park E., Tran K., Miller J.H. (2010). Antibiotic sensitivity profiles determined with an *Escherichia coli* gene knockout collection: Generating an antibiotic bar code. Antimicrob. Agents Chemother..

[15] Tamae C., Liu A., Kim K., Sitz D., Hong J., Becket E., Bui A., Solaimani P., Tran K.P., Yang H. (2008). Determination of antibiotic hypersensitivity among 4,000 single-gene-knockout mutants of *Escherichia coli*. J. Bacteriol..

[16] Alvarez-Ortega C., Wiegand I., Olivares J., Hancock R.E., Martinez J.L. (2010). Genetic determinants involved in the susceptibility of *Pseudomonas aeruginosa* to beta-lactam antibiotics. Antimicrob. Agents Chemother..

[17] Martinez J.L., Rojo F. (2011). Metabolic regulation of antibiotic resistance. FEMS Microbiol. Rev..

[18] Linares J.F., Moreno R., Fajardo A., Martinez-Solano L., Escalante R., Rojo F., Martinez J.L. (2010). The global regulator Crc modulates metabolism, susceptibility to antibiotics and virulence in *Pseudomonas aeruginosa*. Environ. Microbiol..

[19] Allison K.R., Brynildsen M.P., Collins J.J. (2011). Metabolite-enabled eradication of bacterial persisters by aminoglycosides. Nature.

[20] Pethe K., Sequeira P.C., Agarwalla S., Rhee K., Kuhen K., Phong W.Y., Patel V., Beer D., Walker J.R., Duraiswamy J. (2010). A chemical genetic screen in *Mycobacterium tuberculosis* identifies carbon-source-dependent growth inhibitors devoid of *in vivo* efficacy. Nat. Commun..

[21] Lee S.W., Foley E.J., Epstein J.A. (1944). Mode of Action of penicillin: I. Bacterial growth and penicillin activity-*Staphylococcus aureus* FDA. J. Bacteriol..

[22] Mc Dermott W. (1958). Microbial persistence. Yale J. Biol. Med..

[23] Eagle H. (1949). The effect of the size of the inoculum and the age of the infection on the curative dose of penicillin in experimental infections with *Streptococci*, *Pneumococci*, and *Treponema pallidum*. J. Exp. Med..

[24] Bull J.J., Levin B.R., DeRouin T., Walker N., Bloch C.A. (2002). Dynamics of success and failure in phage and antibiotic therapy in experimental infections. BMC Microbiol..

[25] Clement S., Vaudaux P., Francois P., Schrenzel J., Huggler E., Kampf S., Chaponnier C., Lew D., Lacroix J.S. (2005). Evidence of an intracellular reservoir in the nasal mucosa of patients with recurrent *Staphylococcus aureus* rhinosinusitis. J. Infect. Dis..

[26] Fitoussi F., Cohen R., Brami G., Doit C., Brahimi N., de la Rocque F., Bingen E. (1997). Molecular DNA analysis for differentiation of persistence or relapse from recurrence in treatment failure of *Streptococcus pyogenes* pharyngitis. Eur. J. Clin. Microbiol. Infect. Dis..

[27] Toman K. (1981). Bacterial persistence in leprosy. Int. J. Lepr. Other Mycobact. Dis..

[28] McCune R.M., Tompsett R. (1956). Fate of *Mycobacterium tuberculosis* in mouse tissues as determined by the microbial enumeration technique. I. The persistence of drug-susceptible tubercle bacilli in the tissues despite prolonged antimicrobial therapy. J. Exp. Med..

[29] Ginsberg A.M. (2010). Drugs in development for tuberculosis. Drugs.

[30] Koul A., Arnoult E., Lounis N., Guillemont J., Andries K. (2011). The challenge of new drug discovery for tuberculosis. Nature.

[31] Chao M.C., Rubin E.J. (2010). Letting sleeping dos lie: Does dormancy play a role in tuberculosis?. Annu. Rev. Microbiol..

[32] Costerton J.W., Stewart P.S., Greenberg E.P. (1999). Bacterial biofilms: A common cause of persistent infections. Science.

[33] Hall-Stoodley L., Costerton J.W., Stoodley P. (2004). Bacterial biofilms: From the natural environment to infectious diseases. Nat. Rev. Microbiol..

[34] Kolter R., Greenberg E.P. (2006). Microbial sciences: The superficial life of microbes. Nature.

[35] Hansen S.K., Rainey P.B., Haagensen J.A., Molin S. (2007). Evolution of species interactions in a biofilm community. Nature.

[36] Folkesson A., Haagensen J.A., Zampaloni C., Sternberg C., Molin S. (2008). Biofilm induced tolerance towards antimicrobial peptides. PLoS One.

[37] Fux C.A., Costerton J.W., Stewart P.S., Stoodley P. (2005). Survival strategies of infectious biofilms. Trends Microbiol..

[38] Hogan D., Kolter R. (2002). Why are bacteria refractory to antimicrobials?. Curr. Opin. Microbiol..

[39] Lewis K. (2008). Multidrug tolerance of biofilms and persister cells. Curr. Top. Microbiol. Immunol..

[40] Stewart P.S. (2002). Mechanisms of antibiotic resistance in bacterial biofilms. Int. J. Med. Microbiol..

[41] Stewart P.S., Costerton J.W. (2001). Antibiotic resistance of bacteria in biofilms. Lancet.

[42] Drenkard E., Ausubel F.M. (2002). Pseudomonas biofilm formation and antibiotic resistance are linked to phenotypic variation. Nature.

[43] Davies D. (2003). Understanding biofilm resistance to antibacterial agents. Nat. Rev. Drug Discov..

[44] Hoffman L.R., D’Argenio D.A., MacCoss M.J., Zhang Z., Jones R.A., Miller S.I. (2005). Aminoglycoside antibiotics induce bacterial biofilm formation. Nature.

[45] Linares J.F., Gustafsson I., Baquero F., Martinez J.L. (2006). Antibiotics as intermicrobial signaling agents instead of weapons. Proc. Natl. Acad. Sci. USA.

[46] Suci P.A., Mittelman M.W., Yu F.P., Geesey G.G. (1994). Investigation of ciprofloxacin penetration into *Pseudomonas aeruginosa* biofilms. Antimicrob. Agents Chemother..

[47] Corbin A., Pitts B., Parker A., Stewart P.S. (2011). Antimicrobial penetration and efficacy in an *in vitro* oral biofilm model. Antimicrob. Agents Chemother..

[48] Singh R., Ray P., Das A., Sharma M. (2010). Penetration of antibiotics through *Staphylococcus aureus* and *Staphylococcus epidermidis* biofilms. J. Antimicrob. Chemother..

[49] Dong Y., Chen S., Wang Z., Peng N., Yu J. (2013). Synergy of ultrasound microbubbles and vancomycin against *Staphylococcus epidermidis* biofilm. J. Antimicrob. Chemother..

[50] Walters M.C., Roe F., Bugnicourt A., Franklin M.J., Stewart P.S. (2003). Contributions of antibiotic penetration, oxygen limitation, and low metabolic activity to tolerance of *Pseudomonas aeruginosa* biofilms to ciprofloxacin and tobramycin. Antimicrob. Agents Chemother..

[51] Stewart P.S., Davison W.M., Steenbergen J.N. (2009). Daptomycin rapidly penetrates a *Staphylococcus epidermidis* biofilm. Antimicrob. Agents Chemother..

[52] Lewis K. (2005). Persister cells and the riddle of biofilm survival. Biochemistry (Mosc.).

[53] Sternberg C., Christensen B.B., Johansen T., Toftgaard Nielsen A., Andersen J.B., Givskov M., Molin S. (1999). Distribution of bacterial growth activity in flow-chamber biofilms. Appl. Environ. Microbiol..

[54] Huang C.T., Yu F.P., McFeters G.A., Stewart P.S. (1995). Nonuniform spatial patterns of respiratory activity within biofilms during disinfection. Appl. Environ. Microbiol..

[55] Rani S.A., Pitts B., Beyenal H., Veluchamy R.A., Lewandowski Z., Davison W.M., Buckingham-Meyer K., Stewart P.S. (2007). Spatial patterns of DNA replication, protein synthesis, and oxygen concentration within bacterial biofilms reveal diverse physiological states. J. Bacteriol..

[56] Mulcahy H., Charron-Mazenod L., Lewenza S. (2008). Extracellular DNA chelates cations and induces antibiotic resistance in *Pseudomonas aeruginosa* biofilms. PLoS Pathog..

[57] Hoiby N., Bjarnsholt T., Givskov M., Molin S., Ciofu O. (2010). Antibiotic resistance of bacterial biofilms. Int. J. Antimicrob. Agents.

[58] Pamp S.J., Gjermansen M., Johansen H.K., Tolker-Nielsen T. (2008). Tolerance to the antimicrobial peptide colistin in *Pseudomonas aeruginosa* biofilms is linked to metabolically active cells, and depends on the *pmr* and *mexAB-oprM* genes. Mol. Microbiol..

[59] Nalca Y., Jansch L., Bredenbruch F., Geffers R., Buer J., Haussler S. (2006). Quorum-sensing antagonistic activities of azithromycin in *Pseudomonas aeruginosa* PAO1: A global approach. Antimicrob. Agents Chemother..

[60] Giamarellos-Bourboulis E.J. (2008). Macrolides beyond the conventional antimicrobials: A class of potent immunomodulators. Int. J. Antimicrob. Agents.

[61] Wagner V.E., Iglewski B.H. (2008). *Pseudomonas aeruginosa* Biofilms in CF Infection. Clin. Rev. Allergy Immunol..

[62] Fernandez L., Hancock R.E. (2012). Adaptive and mutational resistance: Role of porins and efflux pumps in drug resistance. Clin. Microbiol. Rev..

[63] Pages J.-M., James C.E., Winterhalter M. (2008). The porin and the permeating antibiotic: A selective diffusion barrier in Gram-negative bacteria. Nat. Rev. Microbiol..

[64] Peterson A.A., Hancock R.E., McGroarty E.J. (1985). Binding of polycationic antibiotics and polyamines to lipopolysaccharides of *Pseudomonas aeruginosa*. J. Bacteriol..

[65] Macfarlane E.L., Kwasnicka A., Hancock R.E. (2000). Role of *Pseudomonas aeruginosa* PhoP-phoQ in resistance to antimicrobial cationic peptides and aminoglycosides. Microbiology.

[66] Macfarlane E.L., Kwasnicka A., Ochs M.M., Hancock R.E. (1999). PhoP-PhoQ homologues in *Pseudomonas aeruginosa* regulate expression of the outer-membrane protein OprH and polymyxin B resistance. Mol. Microbiol..

[67] McPhee J.B., Lewenza S., Hancock R.E.W. (2003). Cationic antimicrobial peptides activate a two-component regulatory system, PmrA-PmrB, that regulates resistance to polymyxin B and cationic antimicrobial peptides in *Pseudomonas aeruginosa*. Mol. Microbiol..

[68] Fernández L., Gooderham W.J., Bains M., McPhee J.B., Wiegand I., Hancock R.E.W. (2010). Adaptive resistance to the “last hope” antibiotics polymyxin B and colistin in *Pseudomonas aeruginosa* is mediated by the novel two-component regulatory system ParR-ParS. Antimicrob. Agents Chemother..

[69] Groisman E.A., Kayser J., Soncini F.C. (1997). Regulation of polymyxin resistance and adaptation to low-Mg^2+^ environments. J. Bacteriol..

[70] Gellatly S.L., Needham B., Madera L., Trent M.S., Hancock R.E.W. (2012). The *Pseudomonas aeruginosa* PhoP-PhoQ two-component regulatory system is induced upon interaction with epithelial cells and controls cytotoxicity and inflammation. Infect. Immun..

[71] Hancock R.E.W., Sahl H.-G. (2006). Antimicrobial and host-defense peptides as new anti-infective therapeutic strategies. Nat. Biotechnol..

[72] Rahmati-Bahram A., Magee J.T., Jackson S.K. (1996). Temperature-dependent aminoglycoside resistance in *Stenotrophomonas (Xanthomonas) maltophilia*; alterations in protein and lipopolysaccharide with growth temperature. J. Antimicrob. Chemother..

[73] Manning A.J., Kuehn M.J. (2011). Contribution of bacterial outer membrane vesicles to innate bacterial defense. BMC Microbiol..

[74] Kulp A., Kuehn M.J. (2010). Biological functions and biogenesis of secreted bacterial outer membrane vesicles. Ann. Rev. Microbiol..

[75] Mortimer P.G., Piddock L.J. (1993). The accumulation of five antibacterial agents in porin-deficient mutants of *Escherichia coli*. J. Antimicrob. Chemother..

[76] Cowan S.W., Schirmer T., Rummel G., Steiert M., Ghosh R., Pauptit R.A., Jansonius J.N., Rosenbusch J.P. (1992). Crystal structures explain functional properties of two *E. coli* porins. Nature.

[77] Forst S., Delgado J., Inouye M. (1989). Phosphorylation of OmpR by the osmosensor EnvZ modulates expression of the ompF and ompC genes in *Escherichia coli*. Proc. Natl. Acad. Sci. USA.

[78] Yoshida T., Qin L., Egger L.A., Inouye M. (2006). Transcription regulation of ompF and ompC by a single transcription factor, OmpR. J. Biol Chem.

[79] Mizuno T., Chou M.Y., Inouye M. (1984). A unique mechanism regulating gene expression: Translational inhibition by a complementary RNA transcript (micRNA). Proc. Natl. Acad. Sci. USA.

[80] Chen S., Zhang A., Blyn L.B., Storz G. (2004). MicC, a second small-RNA regulator of Omp protein expression in *Escherichia coli*. J. Bacteriol..

[81] Takayanagi K., Maeda S., Mizuno T. (1991). Expression of *micF* involved in porin synthesis in *Escherichia coli*: Two distinct cis-acting elements respectively regulate *micF* expression positively and negatively. FEMS Microbiol. Lett..

[82] Delihas N., Forst S. (2001). MicF: An antisense RNA gene involved in response of *Escherichia coli* to global stress factors. J. Mol. Biol..

[83] Pratt L.A., Hsing W., Gibson K.E., Silhavy T.J. (1996). From acids to *osmZ*: Multiple factors influence synthesis of the OmpF and OmpC porins in *Escherichia coli*. Mol. Microbiol..

[84] Chubiz L.M., Rao C.V. (2011). Role of the *mar-sox-rob* regulon in regulating outer membrane porin expression. J. Bacteriol..

[85] Nikaido H., Takatsuka Y. (2009). Mechanisms of RND multidrug efflux pumps. Biochim. Biophys. Acta.

[86] Nikaido H. (2009). Multidrug resistance in bacteria. Annu. Rev. Biochem..

[87] Li X.Z., Nikaido H. (2009). Efflux-mediated drug resistance in bacteria: An update. Drugs.

[88] Blair J.M., Piddock L.J. (2009). Structure, function and inhibition of RND efflux pumps in Gram-negative bacteria: An update. Curr. Opin. Microbiol..

[89] Piddock L.J. (2006). Multidrug-resistance efflux pumps—Not just for resistance. Nat. Rev. Microbiol..

[90] Martinez J.L., Sanchez M.B., Martinez-Solano L., Hernandez A., Garmendia L., Fajardo A., Alvarez-Ortega C. (2009). Functional role of bacterial multidrug efflux pumps in microbial natural ecosystems. FEMS Microbiol. Rev..

[91] Saier M.H., Paulsen I.T. (2001). Phylogeny of multidrug transporters. Semin. Cell. Dev. Biol..

[92] Paulsen I.T., Chen J., Nelson K.E., Saier M.H. (2001). Comparative genomics of microbial drug efflux systems. J. Mol. Microbiol. Biotechnol..

[93] Saier M.H., Paulsen I.T., Sliwinski M.K., Pao S.S., Skurray R.A., Nikaido H. (1998). Evolutionary origins of multidrug and drug-specific efflux pumps in bacteria. Faseb. J..

[94] Paulsen I.T. (2003). Multidrug efflux pumps and resistance: Regulation and evolution. Curr. Opin. Microbiol..

[95] Grkovic S., Brown M.H., Skurray R.A. (2001). Transcriptional regulation of multidrug efflux pumps in bacteria. Semin. Cell. Dev. Biol..

[96] Hernandez A., Ruiz F.M., Romero A., Martinez J.L. (2011). The binding of triclosan to SmeT, the repressor of the multidrug efflux pump SmeDEF, induces antibiotic resistance in *Stenotrophomonas maltophilia*. PLoS Pathog..

[97] Ma D., Cook D.N., Alberti M., Pon N.G., Nikaido H., Hearst J.E. (1995). Genes *acrA* and* acrB* encode a stress-induced efflux system of *Escherichia coli*. Mol. Microbiol..

[98] Lin J., Cagliero C., Guo B., Barton Y.W., Maurel M.C., Payot S., Zhang Q. (2005). Bile salts modulate expression of the CmeABC multidrug efflux pump in *Campylobacter jejuni*. J. Bacteriol..

[99] Nikaido E., Yamaguchi A., Nishino K. (2008). AcrAB multidrug efflux pump regulation in *Salmonella enterica* serovar Typhimurium by RamA in response to environmental signals. J. Biol. Chem..

[100] Lee E.H., Shafer W.M. (1999). The *farAB*-encoded efflux pump mediates resistance of gonococci to long-chained antibacterial fatty acids. Mol. Microbiol..

[101] Shafer W.M., Qu X., Waring A.J., Lehrer R.I. (1998). Modulation of *Neisseria gonorrhoeae* susceptibility to vertebrate antibacterial peptides due to a member of the resistance/nodulation/ division efflux pump family. Proc. Natl. Acad. Sci. USA.

[102] Poole K. (2012). Stress responses as determinants of antimicrobial resistance in Gram-negative bacteria. Trends Microbiol..

[103] Miller P.F., Sulavik M.C. (1996). Overlaps and parallels in the regulation of intrinsic multiple-antibiotic resistance in *Escherichia coli*. Mol. Microbiol..

[104] Chen H., Hu J., Chen P.R., Lan L., Li Z., Hicks L.M., Dinner A.R., He C. (2008). The *Pseudomonas aeruginosa* multidrug efflux regulator MexR uses an oxidation-sensing mechanism. Proc. Natl. Acad. Sci. USA.

[105] Fraud S., Poole K. (2011). Oxidative stress induction of the MexXY multidrug efflux genes and promotion of aminoglycoside resistance development in *Pseudomonas aeruginosa*. Antimicrob. Agents Chemother..

[106] Fetar H., Gilmour C., Klinoski R., Daigle D.M., Dean C.R., Poole K. (2011). *mexEF-oprN* multidrug efflux operon of *Pseudomonas aeruginosa*: Regulation by the MexT activator in response to nitrosative stress and chloramphenicol. Antimicrob. Agents Chemother..

[107] Chico-Calero I., Suarez M., Gonzalez-Zorn B., Scortti M., Slaghuis J., Goebel W., Vazquez-Boland J.A. (2002). Hpt, a bacterial homolog of the microsomal glucose-6-phosphate translocase, mediates rapid intracellular proliferation in Listeria. Proc. Natl. Acad. Sci. USA.

[108] Kahan F.M., Kahan J.S., Cassidy P.J., Kropp H. (1974). The mechanism of action of fosfomycin (phosphonomycin). Ann. NY Acad. Sci..

[109] Ripio M.T., Brehm K., Lara M., Suarez M., Vazquez-Boland J.A. (1997). Glucose-1-phosphate utilization by *Listeria monocytogenes* is PrfA dependent and coordinately expressed with virulence factors. J. Bacteriol..

[110] Moreno R., Marzi S., Romby P., Rojo F. (2009). The Crc global regulator binds to an unpaired A-rich motif at the *Pseudomonas putida alkS* mRNA coding sequence and inhibits translation initiation. Nucleic Acids Res..

[111] Moreno R., Martinez-Gomariz M., Yuste L., Gil C., Rojo F. (2009). The *Pseudomonas putida* Crc global regulator controls the hierarchical assimilation of amino acids in a complete medium: Evidence from proteomic and genomic analyses. Proteomics.

[112] Morales G., Linares J.F., Beloso A., Albar J.P., Martinez J.L., Rojo F. (2004). The *Pseudomonas putida* Crc global regulator controls the expression of genes from several chromosomal catabolic pathways for aromatic compounds. J. Bacteriol..

[113] MacGregor C.H., Wolff J.A., Arora S.K., Phibbs P.V. (1991). Cloning of a catabolite repression control (*crc*) gene from *Pseudomonas aeruginosa*, expression of the gene in *Escherichia coli*, and identification of the gene product in *Pseudomonas aeruginosa*. J. Bacteriol..

[114] Brook I. (1989). Inoculum effect. Rev. Infect. Dis..

[115] Soriano F., Ponte C., Santamaria M., Jimenez-Arriero M. (1990). Relevance of the inoculum effect of antibiotics in the outcome of experimental infections caused by *Escherichia coli*. J. Antimicrob. Chemother..

[116] Reguera J.A., Baquero F., Perez-Diaz J.C., Martinez J.L. (1991). Factors determining resistance to beta-lactam combined with beta-lactamase inhibitors in *Escherichia coli*. J. Antimicrob. Chemother..

[117] Reguera J.A., Baquero F., Perez-Diaz J.C., Martinez J.L. (1988). Synergistic effect of dosage and bacterial inoculum in TEM-1 mediated antibiotic resistance. Eur. J. Clin. Microbiol. Infect. Dis..

[118] Martinez J.L., Blazquez J., Baquero F. (1994). Non-canonical mechanisms of antibiotic resistance. Eur. J. Clin. Microbiol. Infect. Dis..

[119] Martinez J.L., Blazquez J., Vicente M.F., Martinez-Ferrer M., Reguera J.A., Culebras E., Baquero F. (1989). Influence of gene dosing on antibiotic resistance mediated by inactivating enzymes. J. Chemother..

[120] Udekwu K.I., Parrish N., Ankomah P., Baquero F., Levin B.R. (2009). Functional relationship between bacterial cell density and the efficacy of antibiotics. J. Antimicrob. Chemother..

[121] Balaban N.Q., Merrin J., Chait R., Kowalik L., Leibler S. (2004). Bacterial persistence as a phenotypic switch. Science.

[122] Levin B.R. (2004). Microbiology: Noninherited resistance to antibiotics. Science.

[123] Bigger J.W. (1944). Treatment of staphylococcal infections with penicillin by intermittent sterilisation. Lancet.

[124] Kussell E., Kishony R., Balaban N.Q., Leibler S. (2005). Bacterial persistence: A model of survival in changing environments. Genetics.

[125] Balaban N.Q. (2011). Persistence: Mechanisms for triggering and enhancing phenotypic variability. Curr. Opin. Genet. Dev..

[126] Hansen S., Lewis K., Vulic M. (2008). Role of global regulators and nucleotide metabolism in antibiotic tolerance in *Escherichia coli*. Antimicrob. Agents Chemother..

[127] Lewis K. (2010). Persister cells. Annu. Rev. Microbiol..

[128] Gefen O., Gabay C., Mumcuoglu M., Engel G., Balaban N.Q. (2008). Single-cell protein induction dynamics reveals a period of vulnerability to antibiotics in persister bacteria. Proc. Natl. Acad. Sci. USA.

[129] Korch S.B., Henderson T.A., Hill T.M. (2003). Characterization of the *hipA7* allele of* Escherichia coli* and evidence that high persistence is governed by (p)ppGpp synthesis. Mol. Microbiol..

[130] Rotem E., Loinger A., Ronin I., Levin-Reisman I., Gabay C., Shoresh N., Biham O., Balaban N.Q. (2010). Regulation of phenotypic variability by a threshold-based mechanism underlies bacterial persistence. Proc. Natl. Acad. Sci. USA.

[131] Keren I., Shah D., Spoering A., Kaldalu N., Lewis K. (2004). Specialized persister cells and the mechanism of multidrug tolerance in *Escherichia coli*. J. Bacteriol..

[132] Dorr T., Vulic M., Lewis K. (2010). Ciprofloxacin causes persister formation by inducing the TisB toxin in *Escherichia coli*. PLoS Biol..

[133] Helaine S., Thompson J.A., Watson K.G., Liu M., Boyle C., Holden D.W. (2010). Dynamics of intracellular bacterial replication at the single cell level. Proc. Natl. Acad. Sci. USA.

[134] Moker N., Dean C.R., Tao J. (2010). *Pseudomonas aeruginosa* increases formation of multidrug-tolerant persister cells in response to quorum-sensing signaling molecules. J. Bacteriol..

[135] Hentzer M., Wu H., Andersen J.B., Riedel K., Rasmussen T.B., Bagge N., Kumar N., Schembri M.A., Song Z., Kristoffersen P. (2003). Attenuation of *Pseudomonas aeruginosa* virulence by quorum sensing inhibitors. EMBO J..

[136] Wu H., Song Z., Hentzer M., Andersen J.B., Molin S., Givskov M., Hoiby N. (2004). Synthetic furanones inhibit quorum-sensing and enhance bacterial clearance in *Pseudomonas aeruginosa* lung infection in mice. J. Antimicrob. Chemother..

[137] Pan J., Bahar A.A., Syed H., Ren D. (2012). Reverting antibiotic tolerance of *Pseudomonas aeruginosa* PAO1 persister cells by (*Z*)-4-bromo-5-(bromomethylene)-3-methylfuran-2(5*H*)-one. PLoS One.

[138] Kim J.S., Heo P., Yang T.J., Lee K.S., Cho D.H., Kim B.T., Suh J.H., Lim H.J., Shin D., Kim S.K. (2011). Selective killing of bacterial persisters by a single chemical compound without affecting normal antibiotic-sensitive cells. Antimicrob. Agents Chemother..

[139] Grant S.S., Kaufmann B.B., Chand N.S., Haseley N., Hung D.T. (2012). Eradication of bacterial persisters with antibiotic-generated hydroxyl radicals. Proc. Natl. Acad. Sci. USA.

[140] Lomovskaya O., Bostian K.A. (2006). Practical applications and feasibility of efflux pump inhibitors in the clinic—A vision for applied use. Biochem. Pharmacol..

[141] Drawz S.M., Bonomo R.A. (2010). Three decades of beta-lactamase inhibitors. Clin. Microbiol. Rev..

[142] Martinez J.L., Rojo F., Vila J. (2011). Are nonlethal targets useful for developing novel antimicrobials?. Future Microbiol..

